# Inducement of semitendinosus tendon regeneration to the pes anserinus after its harvest for anterior cruciate ligament reconstruction-A new inducer grafting technique

**DOI:** 10.1186/1758-2555-4-17

**Published:** 2012-05-20

**Authors:** Hidetaka Murakami, Takashi Soejima, Takashi Inoue, Tomonoshin Kanazawa, Kouji Noguchi, Michihiro Katouda, Kousuke Tabuchi, Megumi Noyama, Hideki Yasunaga, Kensei Nagata

**Affiliations:** 1 Department of Orthopaedic Surgery, Kurume University School of Medicine, 67 Asahi-machi, Kurume City, Fukuoka, 830-0011, Japan

## Abstract

**Purpose:**

To investigate the usefulness of the “inducer grafting” technique for regeneration of the semitendinosus (ST) tendon after its harvest for anterior cruciate ligament (ACL) reconstruction.

**Methods:**

Twenty knees of 20 patients (mean age at the time of surgery, 23.1 years) underwent ACL reconstruction with a double bundle autograft using the ST tendon (7 patients) and the ST + the gracilis (G) tendons (13 patients).

“Inducer grafting” technique

After harvesting the ST tendon, a passing pin with a loop thread is inserted along with the tendon stripper. The passing pin is pulled out from the medial thigh and the loop thread retained. As an inducer graft, the ST tendon branch is used. After the ACL graft has been secured, the inducer graft is sutured to the pes anserinus and the proximal end passed through by pulling the thread out. Then the inducer graft is placed within the tendon canal. The mean follow-up period was 15 months. The presence and morphology of the regenerated ST tendon were examined by MRI. And the isometric hamstring strength was examined at 45°, 90° and 120° of knee flexion.

**Results:**

One month after the operation in all the patients, MRI demonstrated a low-intensity structure at the anatomical location of the ST, at the level of the superior pole of the patella and the joint line, apparently representing the regenerated ST tendon. Four months after the operation, the distal portion of the regenerated ST tendon had reached the pes anserinus in all patients. Twelve months after the operation, the regenerated ST tendon was hypertrophic in 19 of the 20 patients (95%). The isometric knee flexion torque of the ACL-reconstructed limb was significantly lower at 90° and 120° compared with the contralateral limb.

**Conclusion:**

These results suggest that the “inducer grafting” technique is able to improve the regeneration rate of the harvested ST tendon and promote hypertrophy of the regenerated ST tendon, extending all the way to the pes anserinus. However, this technique couldn’t improve the deficits in knee flexion torque after ACL reconstruction.

## Background

Reconstruction of the anterior cruciate ligament (ACL) using autografts is a well-accepted technique. During the last decade, the hamstring graft has been increasingly used because it is associated with relatively low donor-site morbidity in terms of subjective anterior knee pain, and would result in better knee-walking ability [1-4].

In addition to these advantages of a hamstring graft, the semitendinosus (ST) and gracilis (G) tendons have been reported to have the potential to regenerate after harvest. In 1992, Cross et al. [5] reported regeneration of the ST and G tendons after ACL reconstruction with hamstring autografts. They observed that regeneration occurred from the distal cut end of the muscle belly, and magnetic resonance imaging (MRI) revealed that the regenerated tendons had reinserted in the medial popliteal fossa. After this initial report, several authors confirmed that the regenerated tendons became reinserted in a more proximal position on the tibial plateau, i.e., not at their normal anatomic position [6-10].

On the other hand, some researchers have recently reported a decrease of knee flexion torque at deep flexion after ACL reconstruction using the ST or ST + G tendons [11-15], even though the peak knee flexion torque of an ACL-reconstructed knee can recover to at least 90% of the normal value [1,4,16]. Moreover, Tashiro et al. [14] have reported that the reduction of flexion torque at a knee flexion of 70° and beyond is more remarkable in patients who have undergone ACL reconstruction using the ST and G tendons than in those in whom only the ST has been used. This residual hamstring weakness, in general, does not appear to be a significant functional deficit in terms of daily activities. However, in certain sports activities such as gymnastics, wrestling, and judo, which require flexor strength at deep knee flexion with the hip in the extended position, performance can be considerably affected [12,14,17,18].

Several authors have described the factors responsible for a decrease of flexion torque at deep knee flexion after the ST tendon has regenerated [17,19,20]. Makihara et al. [17] suggested that lack of compensation from the semimembranosus muscle (SM) and biceps femoris muscle (BF), atrophy and shortening of the ST, and abnormal re-insertion site of the regenerated ST tendon reduced the knee-flexion torque at over 60° of flexion in patients in whom the ST tendon had been used for ACL reconstruction. When the re-insertion site of the regenerated ST tendon is proximal to the original site, the moment arm is decreased, thus reducing the flexion ability of the knee.

This means that harvesting of the ST tendon causes shortening of the muscle belly and loss of the contraction strength of the ST, while the abnormal re-insertion site of the regenerated ST tendon lowers the ability to transmit muscular strength. To prevent such a postoperative decrease of knee flexion torque, it is important to resolve these factors. We considered that shortening of the muscle belly immediately after harvest of the ST tendon would be unavoidable, but that insertion of the regenerated ST tendon to its anatomically original site (the pes anserinus) would be possible using our “inducer grafting” technique.

**Figure 1:**
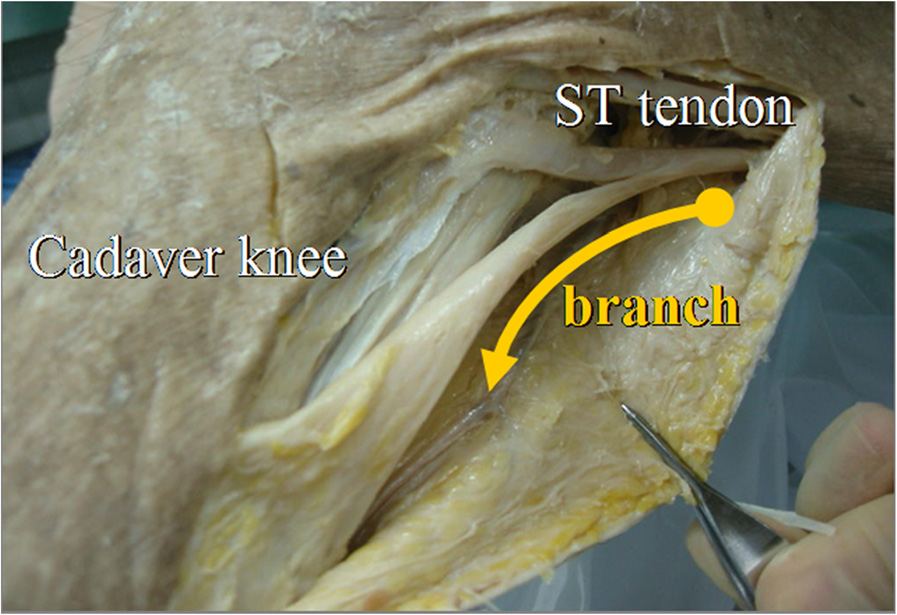
ST tendon and its branch toward the gastrocnemius muscle in a cadaver (right knee).

The purpose of the present study was to assess our “inducer grafting” technique for attaining insertion of the regenerated ST tendon to the pes anserinus and the preparation of the ST branch as “the inducer graft” (Figure 1). Moreover, we investigated the morphology of the regenerated ST tendons obtained using the “inducer grafting” technique and examined isometric knee flexion torque the ACL reconstructed limb and the contralateral normal limb.

## Methods

### Subjects

This new technique was applied to 20 knees of 20 patients who underwent ACL reconstruction using the multi-strand hamstring tendon autograft technique at our institution during a period from November 2005 to November 2008. All the patients had a normal contralateral knee. This study had been approved by the institutional review board and a written informed consent was obtained from all patients before they were included in the study.

There were 11 men and 9 women with a mean age of 23.1 years (range, 15 to 46 years) at the time of the operation. The mean follow-up period was 15 months (range, 12 to 24 months). The ST tendon was used in 7 patients and the ST + G tendons in 13. Among these 20 patients, 2 had a medial collateral ligament injury that was treatable conservatively, and 5 patients had a medial meniscal tear that had been repaired by the inside-out technique. Otherwise, none of the patients had any ligamentous or meniscal injury, or a cartilage lesion of grade 3 severity or more by the Outerbridge classification.

### Surgical technique for ACL reconstruction involving “inducer grafting”

**Figure 2:**
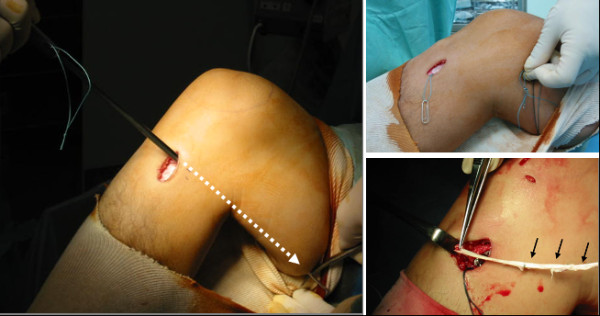
**Inducer grafting procedures: a, A passing pin was guided through the medial thigh along the tendon stripper; b, the passing pin was pulled out of the medial thigh and the loop thread was retained; c, the inducer graft was sutured to the periosteum surrounding the pes anserinus.** (Black arrows; excessively thin and short aponeurosis was augmented using the ST fascia that remained after ACL graft preparation).

A 3-cm longitudinal skin incision was made at the medial side of the tibial tubercle. Through blunt dissection, the pes anserinus was exposed, and the sartorius tendon membrane was split in the direction of the fibers. The ST tendon and its branch toward the gastrocnemius muscle (GC) were then identified, and the branch extending towards the GC was cut as close to the GC as possible. Then, the distal end of the ST tendon was peeled off together with a periosteum flap, and passed through a tendon stripper (Smith & Nephew Inc. Endoscopy, Andover, MA, USA). The stripper was pushed proximally to separate the ST tendon from the muscle by blunt force. However, the stripper was not removed from the tendon canal after the tendon had been pulled out. Next, a passing pin (Smith & Nephew Inc. Endoscopy) with a loop thread was inserted along with the tendon stripper. The passing pin was pulled out from the medial thigh and the loop thread retained (Figure 2a, b). When the ST tendon was less than 24 cm long, we additionally harvested the G tendon in the same manner, but without using the passing pin to insert the loop thread.

**Figure 3:**
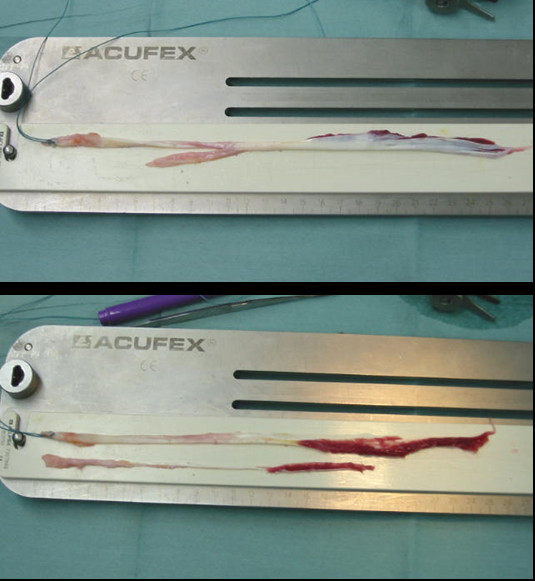
Preparation of the inducer graft: a, removal of the ST tendon connecting the GC branch; b, the ST tendon and its detached branch.

Thereafter, the surgeon performed the double-bundle ACL reconstruction [21] and additional procedures. During ACL reconstruction an assistant surgeon separated the harvested ST tendon and its GC branch (Figure 3a, b). Since the GC branch was connected to the aponeurosis as the superficial layer of the ST tendon proximally, the separation step involved only detachment of the thin aponeurosis from the ST tendon. Thus, the graft for ACL reconstruction was well preserved in terms of both quality and quantity. If the GC branch or the aponeurosis was too thin and short, we were able to augment it using the ST fascia that remained after ACL graft preparation (Figure 2c). We named the branch obtained in this manner the “inducer graft” because the graft was used to induce the regeneration of the ST tendon. In general, the inducer graft was 5 to 6 cm shorter than the harvested ST tendon and 1 to 2 mm in diameter at its narrowest part.

The main portion of the ST tendon was prepared in the usual manner as that for a double-bundle ACL reconstruction, being passed through the bone tunnel from the tibia to the femur. The ACL graft was then fixed to the femur with an Endobutton CL® (Smith & Nephew Inc. Endoscopy), and to the tibia with a double spike plate and screw (Smith & Nephew Inc. Endoscopy).

Before closing the wound after ACL reconstruction, the distal end of the inducer graft was sutured to the periosteum surrounding the pes anserinus (Figure 2c). Then the proximal end of the inducer graft was passed through a loop of the previously retained loop thread, and the latter was pulled out with the knee in extension. In this manner, the inducer graft was placed within the tendon canal after removing the ST tendon. Thereafter, care was taken to avoid any movement of the knee until the wound had been closed and dressed.

### Postoperative rehabilitation

Postoperative rehabilitation was conducted in accordance with our standard protocol for ACL reconstruction without “inducer grafting”. After the knee had been immobilized for 10 days in a brace at 30° of flexion, range of motion exercises were initiated using a continuous passive motion device. Weight-bearing was permitted 2 weeks after the operation, and full weight-bearing was started after 4 weeks. Jogging was permitted after 4 months, and sports activities were allowed from 6 –10 months after the operation.

### MRI examination

MRI images were acquired on a SIGNA Horizon LX 1.5-T magnet (General Electric Medical Systems; Milwaukee, WI, USA) using a 6.5-in extremity coil (Knee Array Coil, General Electric Medical Systems). In each case, the transaxial sequences from the superior pole of the patella to a site 3 cm distal to the tibial tubercle were obtained by T2-weighted imaging 1 month (n = 18), 2 months (n = 16), 4 months (n = 16), and 12 months (n = 20) after the operation. Knees were held in full extension to obtain the images. In the transaxial sequence over the knees, the slice thickness was 4 mm, the repetition time 4000 ms, and the echo time 82.5 ms in the T2-weighted sequence. The presence and the morphology of the regenerated ST tendon at the level of the superior pole of the patella, the joint line, and the pes anserinus were evaluated in the transaxial sequence.

The cross-sectional area (CSA) of the ST tendons was compared between the preoperative images and those obtained at 12 months after the operation. In each case the CSA of the native ST tendon and regenerated ST tendon were measured at the level of the joint line using ImageJ software (version 1.40 g, National Institutes of Health, USA). The MRI scans were reviewed by three orthopaedic surgeons (HM, TS and TK). The reviews were performed independently and any discrepancies were settled by a consensus within the group.

### Knee flexion torque measurement

Isometric knee flexion torque was measured at 12 months after the operation using the instrumented manual muscle testing of Elmlinger’s methods [15]. Instrumented manual muscle testing of isometric knee flexion torque at 45°, 90° and 120°knee flexion was performed with neutral tibial rotation by micro-FET digital dynamometer (HOGGAN Health Industries, Inc., UT, USA). During prone position isometric testing, a canvas strap was secured across the patient’s hips to prevent hip flexion. The contralateral normal limb knee was tested before the ACL reconstructed limb. Isometric torque was measured 3 times, with the average value used for the evaluation.

### Statistical analysis

A paired Student’s t test was used to test for differences in CSA and isometric knee flexion torque. Statistical significance was established at P < 0.05. Statistical analysis was performed using Windows JMP 7.0 software (SAS, Cary, NC, USA).

## Results

The mean length of the GC branch of the harvested ST tendon was 4.5 ± 2 cm. After the GC branch had been separated from the ST tendon itself, an inducer graft, 20.7 ± 5 cm long on average, was obtained. The mean diameter of the graft was 1.9 mm at 10 cm proximal from the end suture to the pes anserinus.

**Figure 4:**
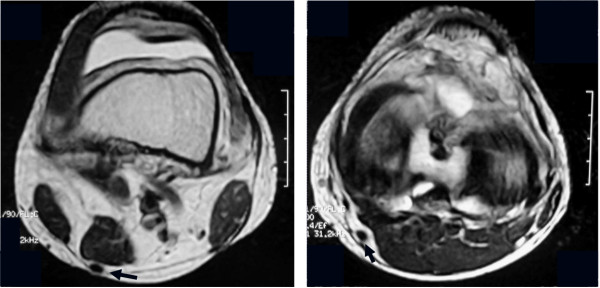
One month after the operation, a low-intensity structure was observed at the same position of the preoperative ST tendon at the level of the superior pole of the patella (a) and the joint line (b).

One month after the operation, MRI images revealed a structure of low intensity with a non-homogeneous interior at the anatomical location of the original ST tendon, at the level of the superior pole of the patella and the joint line (Figure 4a, b). However, at the level of the pes anserinus, a low-intensity structure with a non-homogeneous interior was noted on MRI images in 10 of 18 patients (55.6%). Two months after the operation, low-intensity structure at the level of the superior pole of the patella and the joint line became thicker.

**Figure 5:**
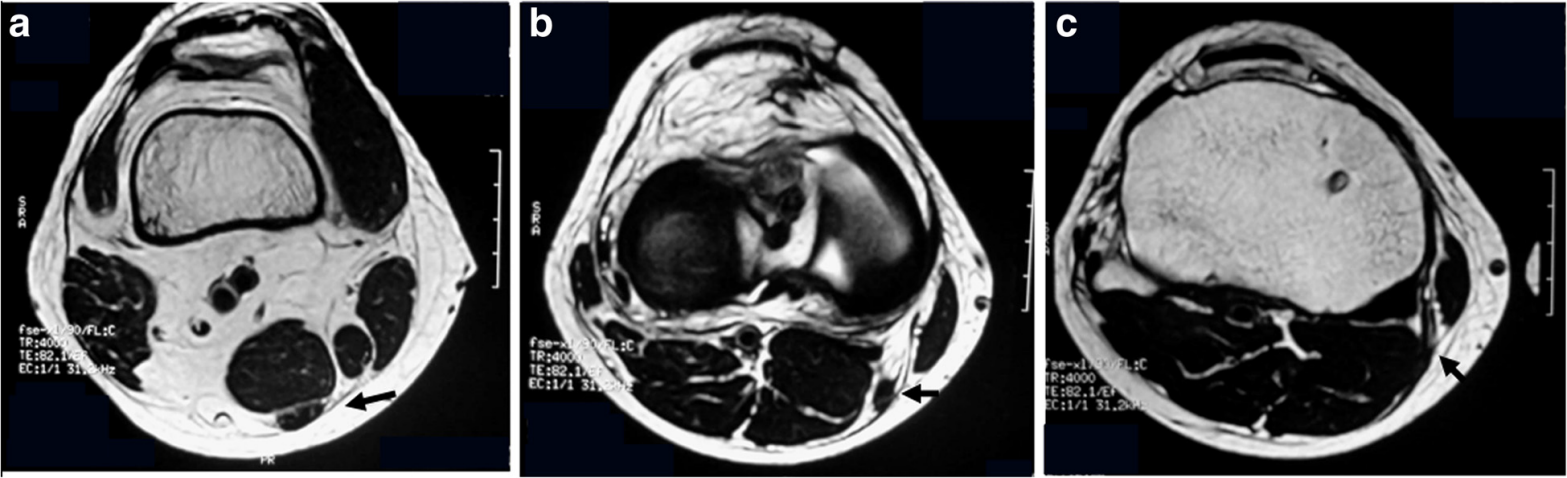
The regenerated ST tendon had reached the pes anserinus 4 months after the operation at the level of the superior pole of the patella (a), the joint line (b) and the pes anserinus (c).

Four months after the operation, a homogeneous, low-intensity structure was detected at the level of the superior pole of the patella, the joint line, and the pes anserinus in all 16 patients, and it was confirmed that its distal portion had reached the pes anserinus (Figure 5a, b and c).

**Figure 6:**
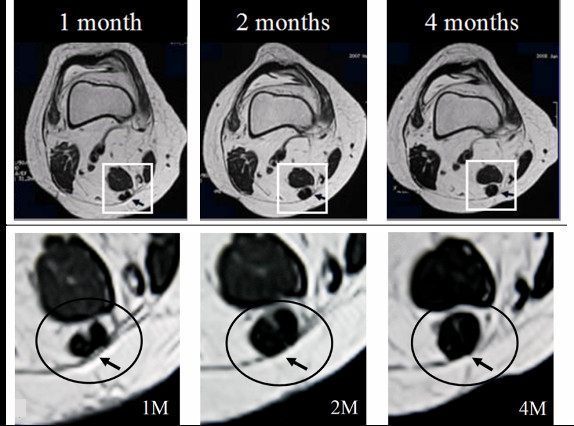
**The changes observed on consecutive MRI at the level of the superior pole of the patella.** MRI performed at 1, 2 and 4 months after the operation (**a**). The two round regions of low intensity were enlarged and connected by 2 months, and were unified by 4 months after the operation (**b**).

Furthermore, it was possible to capture consecutive morphologic changes at the level of the superior pole of the patella, from 1 month to 4 months after the operation in 3 patients. At one month, two round, low-intensity areas were identified. At 2 months after the operation, these areas were larger and connected, and by 4 months had become unified into a homogeneous low-intensity area (Figure 6a, b).

**Figure 7:**
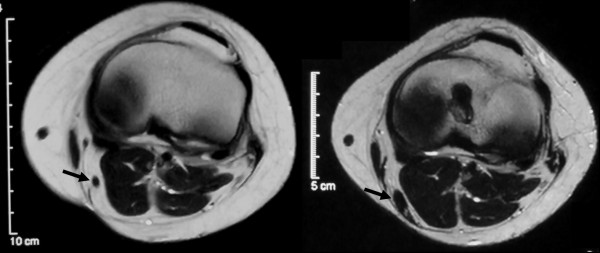
The regenerated ST tendon appeared to be hypertrophic at the level of the joint line. a, before the operation; b, 12 months after the operation.

At 12 months after the operation, the homogeneous low-intensity structure had become clearer than at 4 months. The CSA of the ST tendons was compared between the preoperative images and those obtained at 12 months after surgery. Overall, the average CSA of the regenerated ST tendons was 0.31 ± 0.28 cm², whereas the preoperative value was 0.12 ± 0.04 cm². Thus, the CSA of the regenerated ST tendon obtained using an inducer graft was significantly thicker than the native ST tendon (P = 0.008). Based on the difference in the measured value, the regenerated tendons were divided into two groups, being hypertrophic in 19 (95%) of the 20 patients and atrophic in the remaining 1 (5%), relative to the native ST tendon (Figure 7a, b).

The isometric knee flexion torque of the ACL reconstructed limb was significantly lower at 90° and 120° compared with the contralateral limb, whereas it was not significant at 45° (Table 1).

**Table 1:** Isometric Knee Flexion Torque (N-m/kg)

	**Knee flexion angle**		
	**45°**	**90°**	**120°**
Nonoperated side	2.7 ± 0.8	2.1 ± 0.8	1.2 ± 0.7
Operated side	2.7 ± 0.9	1.37 ± 0.5 *	0.67 ± 0.1 *

* A significant difference was observed between the operated and nonoperated knees (P < 0.05).

## Discussion

The regenerative potential of the harvested hamstring tendon has recently been reported. However, it has been noted that the regenerated ST tendon becomes attached at an abnormal site [5-10].

Furthermore, studies evaluating postoperative hamstring muscle performance according to various parameters have shown that knee flexion torque at a deep angle of knee flexion is reduced after ACL reconstruction using the hamstring tendons [11-15]. Makihara et al. [17] suggested that the lack of compensation from the SM and BF, the atrophy and shortening of the ST, and the abnormal re-insertion site of the regenerated ST tendon all play a role in the reduction of knee flexion torque at angles of over 60° in patients who have undergone ACL reconstruction using the ST tendon. Nishino et al. [19] suggested that the insertion sites of flexor tendons in the knee, including the ST, affect the flexion moment arm that contributes to the production of knee-flexion torque.

Such studies indicate that the harvested ST tendon can regenerate, but does not have sufficient muscular strength. Namely, the regenerated ST tendon cannot function normally as a muscle-tendon complex. Therefore, we developed the “inducer grafting” technique, which stimulates regeneration of the ST tendon, and in turn induces the pes anserinus.

Our MRI findings at 1 month after the operation revealed a structure showing non- homogeneous low-signal intensity, considered to be the regenerated ST tendon, at the superior pole of the patella and the joint line in all of the patients. However, at the level of the pes anserinus a low-signal intensity structure was seen in 10 of the 18 patients (55.6%).

Previous reports of natural regeneration cases described results that were different from ours. Rispoli et al. [22] reported that a low-signal-intensity structure with the morphologic appearance of a normal tendon was evident only at the level of the superior pole of the patella even at the 6th week, and that the structure was ill-defined below the level of the mid-patella and blended into the adjacent fascia. The appearance of low-intensity structure in our cases was earlier than the previous report. That was suggested that the inducer graft could stimulate the regeneration of ST tendon at an early stage after the operation.

Four months after the operation, we found that the regenerated ST tendon extended toward the pes anserinus in all patients and we also identified the regenerated ST tendon in all 20 (100%) at 12 months. In a prospective MRI study, Eriksson et al. [23] reported a regeneration incidence of 73% (8/11) at 6–12 months after tendon harvest. And in our previous MRI study [24] we investigated the regeneration incidence of the ST tendon in 2 groups without the inducer graft at a mean follow-up of 17 months after ACL reconstruction. The ST tendon harvested group was 73% (22/30 cases) and the ST + G tendons harvested group was 67% (20/30 cases) at the joint line.

Tadokoro et al. [18] observed the regenerated ST tendon on MRI scans in 22 (78.6%) of 28 patients more than 2 years after ACL reconstruction. They divided the regenerated ST tendons into hypertrophic, atrophic, and unidentifiable types by comparison with the CSA on the uninvolved side, and detected 6 (21.4%) hypertrophic, 16 (57.1%) atrophic and 6 (21.4%) unidentifiable tendons. In the present study, 19 (95%) of our 20 patients showed hypertrophic regeneration at 12 months after the operation, and 1 patient (5%) showed tendons that were atrophic in comparison with the MRI findings before surgery. The “inducer grafting” technique was able to improve the regeneration rate of the harvested ST tendon and promote hypertrophy of the regenerated ST tendon.

With regard to the mechanisms of ST tendon regeneration, Eriksson et al. [25] hypothesized that hematoma in the tendon canal after tendon harvest might act as a scaffold for the infiltration of fibroblast precursor cells. Rispoli et al. [22] speculated that regeneration occurred in a proximal to distal manner along the fascial planes. The normal G and ST tendons lie in the fascia of the medial aspect of the knee. This fascia forms a 3- to 4-cm band around the tendons 8 to 10 cm proximal to the pes anserinus. Furthermore, Okahashi et al. [26] speculated that the stripping procedure for removal of the tendon would leave synovial cells, which possess the ability to differentiate, and that the mechanical stress generated by motions such as rehabilitation might induce differentiation of the tendon. Carofino [20] suggested that in cases where only the ST tendon is harvested leaving the G intact, the latter may assist tracking of the regenerating structure to the pes anserinus and facilitate anatomic regeneration. In the present study, we expected the inducer graft to act as 1) a scaffold for cell infiltration, and 2) a guide for the tendon canal toward the pes anserinus along the fascial planes.

Irie et al. [27] reported that the level of the ST and G muscle stumps after tendon harvesting were an irregular variance. Six of 12 ST muscle stumps remained beyond the joint line level, and 4 did not reach the joint line level. Three ST muscle stumps were located more than 25 mm proximal to the joint line level.

In the present study 3 patients were able to visualize the consecutive morphologic changes of the same ST stump level at an early stage after the operation. MRI images showed two low-intensity areas adjacent each other at the level of the superior pole of the patella 1 month after the operation. At 2 months after the operation, these areas were larger and connected, and were unified into a homogeneous low-intensity area at 4 months. Such consecutive changes demonstrated by MRI may indicate that regeneration of the ST tendon distally from the proximal cut end allows connection with the inducer graft, with subsequent gradual fusion and unification of the two structures.

More recently, Ferretti et al. [28] reported a clinical study that focused on the anatomical attachment of the regenerated ST tendon. They utilized a modified harvesting technique in which the ST tendon was cut 4 cm proximal to its tibial insertion site and its proximal end was then sutured to the sartorius fascia as a guide for regeneration of the ST tendon toward the pes anserinus. They had to alter the reconstruction method in 2 of 19 patients because the length of the graft was insufficient for ACL reconstruction. Among the 17 patients whose ACL was reconstructed successfully, MRI demonstrated attachment of the regenerated ST tendon to the posteromedial corner of the tibial plateau in 5 (29%). In the present study, ACL reconstruction was performed without any problems in 20 patients; the ST tendon was regenerated 4 months after the operation, and its attachment end reached the pes anserinus. In comparison with the “guide” used by Ferretti et al. [28], the inducer graft we devised was longer and reached the inside of the tendon canal. It did not affect ACL reconstruction, and was thus likely to be more useful.

From the instrumented manual muscle testing [15], the isometric knee flexion torque of the ACL reconstructed limb using the “inducer grafting” technique at 45° was not significantly different than that of the contralateral normal limb. However, at 90° and 120°, the torque of ACL reconstructed limb was significantly lower than normal.

Makihara et al. [17] suggested that the ST played an important role at over 60° of knee flexion, while the SM and BF worked together to produced the peak knee flexion torque occurring at less angles. Therefore, the “inducer graft” technique could not improve the deficits in knee flexion torque of the regenerated ST tendon after ACL reconstruction.

There were several limitations to our study. The first was that we did not examine the micro- and macroscopic features by biopsy or 3-dimensional (3D) computed tomography (CT) examination [29,30] at the site of attachment of the regenerated ST tendon. Nakamura et al. [29] considered that the 3D CT technique might be helpful for investigating the full-length morphology of the regenerated tendon at the harvest site. The volume-rendered 3D image provided by multidetector CT allows more detailed and solid geometric visualization of soft tissue than conventional MRI. Our second study limitation was related to the surgical procedure; insertion of the passing pin that holds the tendon stripper and passing it through the skin of the medial thigh was difficult. Although there was no infection, deep thrombosis, nerve or arterial injury, or other complications in this series, the development of instruments specifically for this purpose would be desirable in order to shorten the operation time.

Since the regenerated ST tendon has been reported to be mature by 18 months after surgery [6], long-term studies involving a larger number of patients will be needed to clarify issues such as the mechanical properties of the regenerated ST tendon created using the inducer graft. Moreover, it will be necessary to investigate the mechanisms of ST tendon regeneration when using the inducer graft by means of an animal model [10]. In view of the functional ability of the regenerated ST tendon and its potential for reharvest [31], some factors such as shortening of the muscle belly of the regenerated ST must be resolved.

## Conclusion

The results of present study suggest that the “inducer grafting” technique can improve the regeneration rate of the harvested ST tendon and promote hypertrophy of the regenerated ST tendon, as well as inducing extension of the regenerated ST tendon to the pes anserinus within a short time after ACL reconstruction using a hamstring autograft. However, this technique couldn’t improve the deficits in knee flexion torque after ACL reconstruction.

## Competing interests

The authors declare that they have no competing interests.

## Authors’ contributions

HM, TS, HY and KN have contributed to the conception design, data collection, interpretation, and drafting of the manuscript. TI, TK, KN, MK, KT and MN contributed to the collection and analyze of the data. HM, TS and KT contributed to the review of the MRIs and calculated knee flexion torque values. All authors have read and approved the final manuscript.
